# Can Cognitive Activities during Breaks in Repetitive Manual Work Accelerate Recovery from Fatigue? A Controlled Experiment

**DOI:** 10.1371/journal.pone.0112090

**Published:** 2014-11-06

**Authors:** Svend Erik Mathiassen, David M. Hallman, Eugene Lyskov, Staffan Hygge

**Affiliations:** 1 Centre for Musculoskeletal Research, Department of Occupational and Public Health Sciences, University of Gävle, Gävle, Sweden; 2 Department of Building, Energy and Environmental Engineering, University of Gävle, Gävle, Sweden; Tokyo Institute of Technology, Japan

## Abstract

Neurophysiologic theory and some empirical evidence suggest that fatigue caused by physical work may be more effectively recovered during “diverting” periods of cognitive activity than during passive rest; a phenomenon of great interest in working life. We investigated the extent to which development and recovery of fatigue during repeated bouts of an occupationally relevant reaching task was influenced by the difficulty of a cognitive activity between these bouts. Eighteen male volunteers performed three experimental sessions, consisting of six 7-min bouts of reaching alternating with 3 minutes of a memory test differing in difficulty between sessions. Throughout each session, recordings were made of upper trapezius muscle activity using electromyography (EMG), heart rate and heart rate variability (HRV) using electrocardiography, arterial blood pressure, and perceived fatigue (Borg CR10 scale and SOFI). A test battery before, immediately after and 1 hour after the work period included measurements of maximal shoulder elevation strength (MVC), pressure pain threshold (PPT) over the trapezius muscles, and a submaximal isometric contraction. As expected, perceived fatigue and EMG amplitude increased during the physical work bouts. Recovery did occur between the bouts, but fatigue accumulated throughout the work period. Neither EMG changes nor recovery of perceived fatigue during breaks were influenced by cognitive task difficulty, while heart rate and HRV recovered the *most* during breaks with the most difficult task. Recovery of perceived fatigue after the 1 hour work period was also most pronounced for the most difficult cognitive condition, while MVC and PPT showed ambiguous patterns, and EMG recovered similarly after all three cognitive protocols. Thus, we could confirm that cognitive tasks between bouts of fatiguing physical work can, indeed, accelerate recovery of some factors associated with fatigue, even if benefits may be moderate and some responses may be equivocal. Our results encourage further research into combinations of physical and mental tasks in an occupational context.

## Introduction

Introducing breaks is a key intervention to provide recovery after fatiguing physical work. Finding an optimal distribution across time of work periods and breaks has been a challenge in physiologic and ergonomics research for almost one century [1]–[6], and has also engaged management scientists [7], [8]. Rest breaks have unambiguously been shown to alleviate fatigue in isometric, isotonic contractions, while the effect of added breaks in occupational work has been less obvious [9], even if recent theories explaining the origin and persistence of musculoskeletal disorders suggest that total rest will be a prerequisite for obtaining recovery of certain low-threshold “Cinderella” motor units that remain recruited as long as the muscle is active [10], [11].

The basic rationale in ergonomics and management science for introducing breaks in occupational work is to maintain good productivity and a sustainable health and well-being of the individual worker by providing an opportunity to recover from tasks that might otherwise lead to a loss in performance [12]. In recent years, breaks have been explained and discussed as one option among others of how to obtain optimal variation in a job rather than as an initiative to specifically provide rest [9], [13]. In line with this view, some occupational studies have investigated alternative non-rest “break” activities, including productive tasks. A majority of these studies have been devoted to physically “active” breaks, based on the notion that *more* activity will be a more effective source of variation than rest in occupations characterized by low-level, long-lasting muscle activity, such as office work or light industrial assembly [14]–[17].

Dating back more than 100 years and inspiring neurophysiological research since then, Sechenov (later cited in [18]) proposed that any type of “diverting” activity – physical as well as mental – would positively influence recovery from fatigue, the reason being that powerful processes leading to fatigue (in the sense of a decreased performance capacity) reside in the central nervous system and thus can be influenced by “diverting” central brain processes. As suggested in more recent studies, a mental activity may more effectively enhance recovery if it activates certain regions in the brain known to be involved in central fatigue processes, i.e. primarily the motor and prefrontal cortex [19]. Central fatigue processes add to and interact with peripheral physiologic responses locally in the exercising muscles, such as changes in the chemical environment [20], [21], that may lead to a decreased force-generating capacity [22], [23].

Following the ideas of Sechenov, “active” breaks with an organized mental activity between bouts of physical work might be more effective in alleviating fatigue than rest breaks without a focussed mental activation. This may appear a challenging and even awkward notion, considering that several studies have shown that mental tasks practiced “*on top*” of a physical work task may lead to increased muscle activity [24]–[29], and to more pronounced fatigue than the physical task alone [30], [31], even if these results have been contested in other studies [32]–[34]. However, the effect of breaks with an organized mental activity between bouts of physical work has, to our knowledge, been empirically pursued only in very few studies. This is surprising, considering that alternating physical and mental work occurs in many occupations, and that organized breaks are probably often spent at activities that differ from rest and include cognitive engagement [35]. In one study of repeated elbow or finger flexor contractions, Asmussen and Mazin [36] found that the amount of work that could be performed until exhaustion was larger after a break containing mental arithmetic than after a passive rest break. As the authors did not observe any differences in muscle blood flow between active and passive breaks, they concluded that the mentally active breaks influenced recovery to a major extent via mechanisms in the central nervous system. In the 2002 Volvo Award in Biomechanics study, Davis and colleagues [37] showed that kinematics and muscle activation in the lower back during lifting was influenced by a preceding “complex” mental task (reading and interpreting an 8-digit number), particularly at a high lifting frequency. Thus, the biomechanical exposure was *larger* after the complex mental load than after a “simple” mental task involving an easy verbal command; the study did not explicitly evaluate recovery. Finally, in a recent study of repeated maximal isokinetic knee extensions, Stock and colleagues [38] found, in line with the results by Asmussen and Mazin, that a mental task (mathematical problem-solving) accelerated recovery between two exercise bouts as compared to quiet rest; a diverting physical task had the same positive effect. Thus, Stock and colleagues [38] agree with Asmussen and Mazin [39] in suggesting that the mental task most probably exerts its effect through processes in the central nervous system.

While a reasonable expectation would be that the possible ability of a mental activity to accelerate recovery depends on the specific combination of alternating physical and mental tasks, as well as the time pattern of alternation [31], [34], the cited studies on serial combinations of physical and mental activity differ considerably in both physical and mental task protocols. Two of the studies used physical tasks requiring maximal effort, i.e. repeated maximal force exertions [38] or continuation of the task until exhaustion [39], which may be of limited validity as a model of occupational work. The study by Davis and colleagues did have an occupational basis, but investigated a lifting task. Thus, no evidence is available on effects of mental activity in combinations involving repetitive, sub-maximal work with the upper extremity, as in industrial assembly or cashier's work. Only the study by Davis and colleagues [37] addressed different levels of difficulty of the mental task, which can reasonably be expected to also influence its effect on recovery [28]. Furthermore, none of the three studies were devoted to multiple cycles of alternating physical and mental activity, and none of them followed physiologic responses using a comprehensive set-up of methods reflecting both central and peripheral factors of relevance to fatigue.

The present study therefore aimed at investigating the extent to which cognitive activities of different difficulty influence the development and recovery of fatigue resulting from repeated bouts of a repetitive, occupationally relevant reaching task in a population of young males.

## Methods

### Subjects

Eighteen healthy male volunteers aged 20–34 years (mean 24.2 (SD 4.3)), with BMI 18.6–29.0 kg·m^−2^ (mean 23.7 (SD 3.0)) participated in the study. Only males were recruited so as to avoid possible gender effects on the responses to the studied physical and mental task. The subjects answered to a public poster recruitment campaign at the University of Gävle, and entered into the study after providing their written consent, on the basis of information about the aim and experimental procedures of the study. The subjects had no self-reported history of major trauma to the back, arms, neck and shoulders, nor of diseases which could affect motor function or the autonomic nervous system; they did not use any medication regularly; and they reported to be free of upper extremity pain, fatigue and other signs of discomfort at the time of the study.

The study was approved by the Ethical Review Board in Uppsala, Sweden, and conducted in agreement with the Helsinki declaration.

### Protocol

The study comprised three experimental sessions separated by at least one week. All three sessions were performed in a laboratory at a constant ambient temperature of 22°C. With very few exceptions, all sessions for any particular subject began at the same time of the day, selected according to the subject's preferences. On a separate day prior to the first session, subjects carried out a training and familiarisation trial. Training continued until the subject reached a stable work rhythm, or for a minimum of 3 minutes, corresponding to about 90 work cycles (see below).

**Figure 1:**
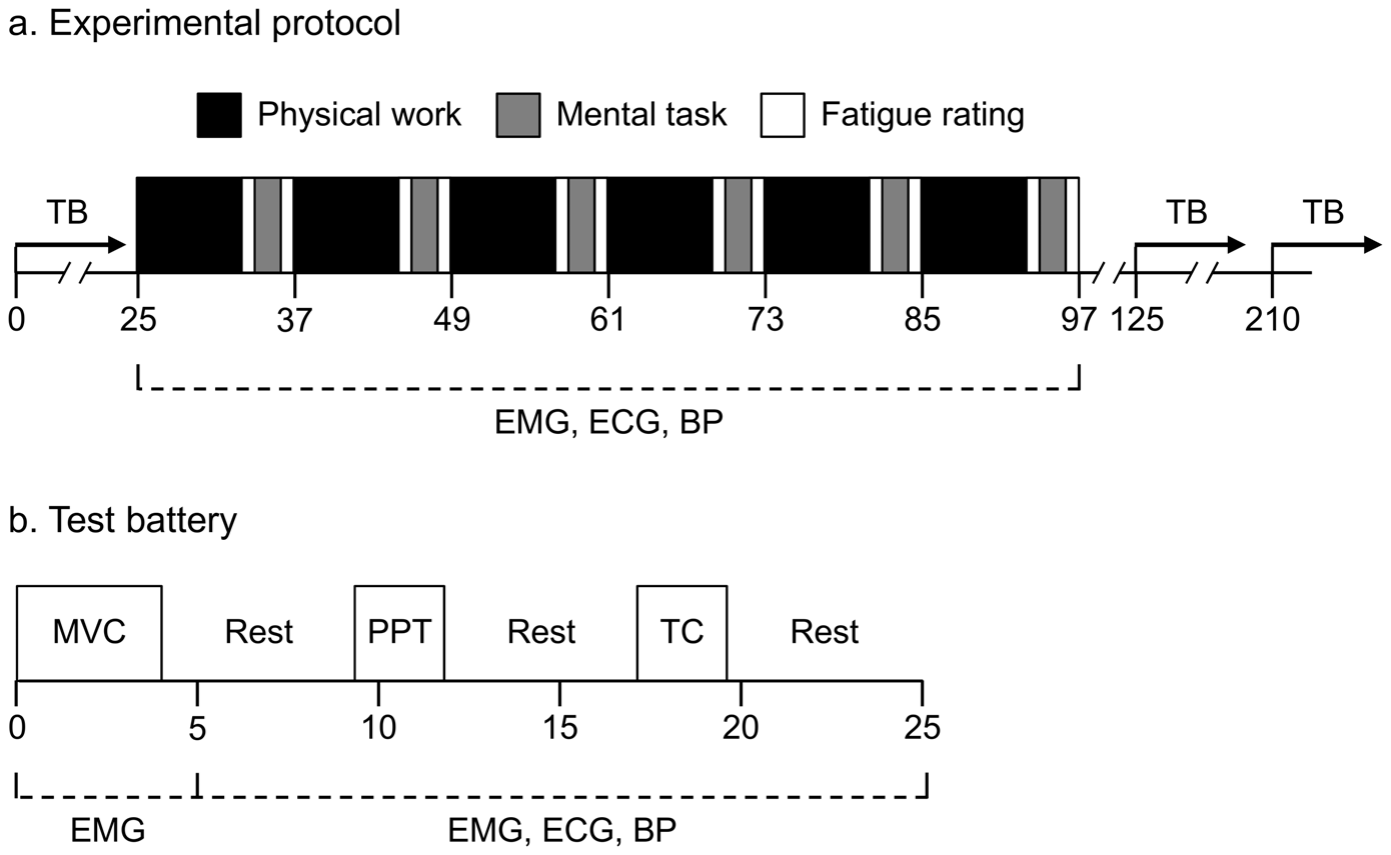
Experimental protocol and test battery. The protocol (Figure 1a) included six 7-minute bouts of a repetitive reaching task, interspersed by 5-minute breaks comprising two ratings of perceived fatigue and a 3-minute mental task. A test battery (TB, Figure 1b) was performed before, immediately after, and one hour after the work period. Both x-axes show time in minutes. EMG, electromyography; ECG, electrocardiography; BP, blood pressure; MVC, maximal voluntary contraction; PPT, pressure pain threshold; TC, test contraction.

Subjects were instructed to avoid smoking and caffeine intake thirty minutes before each experimental session, meals two hours before and intensive physical exercise two days before. During each session, subjects performed six bouts of a repetitive reaching task (see below) interrupted by rest breaks (Figure 1). During these breaks, subjects performed a mental task (a working memory test; see below) with three levels of cognitive difficulty, one in each experimental session. The order of sessions with different difficulties was balanced between subjects.

### Physical task

The physical work task consisted of repeated movements of a 300 g manipulandum held in the right (dominant) hand, pushing two “near” and “far” buttons at 1 Hz (one complete cycle lasting 2 s) as guided by a metronome [40]. The distance between the two buttons were adjusted according to each subject's arm length so that the angle of the elbow was 90° and 160° when pressing the near and far buttons, respectively. The subject was seated comfortably at the work station in an adjustable chair with a knee angle of approximately 90°, the left arm resting on the table. A detailed description of the task is provided in a previous publication devoted specifically to fatigue effects on motor control during repetitive work, without any attention being paid to effects of mental tasks [41].

### Mental task

During the breaks between physical work bouts, the subject performed a mental task (MT) often referred to as running memory span [42]. Eight series of 8–11 letters were presented on a computer screen in front of the subject. Letters were presented once per second with 0.8 s exposure time and 0.2 s of black screen. At the three levels of cognitive difficulty, the subject was asked to recall and orally report the last (MT1), two last (MT2) or three last (MT3) letters, respectively. MT1 was included to represent a condition in which the subject would have to focus his cognitive, visual, and motor attention on a similar activity as that eventually presented in the more difficult mental conditions MT2 and MT3, while at the same time being so easy that it could readily be accomplished by all subjects. Thus, MT1 represents a more consistent reference for evaluating the effects of MT2 and MT3 than the more common choice of passive rest as a control condition, during which the attention of subjects may differ widely in both focus and intensity. The subject's answer was scored by the examiner in terms of the number of correctly recalled letters in the correct order. The subject did not receive any feed-back on performance. Analyses after the full experiment confirmed that the three mental task conditions differed significantly in cognitive demands (ANOVA, F = 88.17; p<0,001); MT3 was associated with a markedly reduced performance (83% correct answers) compared to MT2 (95%) and MT1 (99%).

### Physiological and psychophysical measurements

#### Ratings of perceived fatigue

Before and after each of the six physical work bouts, the subject was asked to rate his perceived fatigue in the neck and shoulders on the 10-grade Borg CR10 scale [43], and to respond to items from the Swedish Occupational Fatigue Inventory (SOFI; [44]). SOFI measures, on a scale from 0 to 6, perceived fatigue in five dimensions, i.e. *lack of energy*, *physical exertion*, *physical discomfort*, *lack of motivation* and *sleepiness*. In the present study, a short version of SOFI was used, consisting of five items (*spent*, *breathing heavily*, *aching*, *uninterested* and *sleepy*), which have been shown to strongly represent each of the five fatigue dimensions [44].

#### Electromyography (EMG)

EMG was recorded bilaterally from the upper trapezius muscles using pairs of self-adhesive surface electrodes with 20 mm inter-electrode distance, centres placed 2 cm lateral to the midpoint between vertebrae C7 and the acromion [45]. The trapezius muscle was selected as is standard in occupational studies [45]–[47], so as to represent a region often associated with work-related pain and discomfort [48]. The signal was amplified, band-pass filtered at 10–1000 Hz and sampled at 2000 Hz. Off-line, after cleaning the signal from obvious artefacts, it was root mean square (RMS) converted using consecutive 250 ms windows. The EMG RMS amplitude was then normalised to the mean amplitude during the test contraction in the baseline test battery (see below; [45]), and expressed in percent of reference voluntary exertion (%RVE). The normalized EMG amplitude and the raw EMG Mean Power Frequency (MPF) during the entire first and last minute of each physical work bout were retrieved for further analysis.

#### Electrocardiography (ECG)

ECG was recorded from the thorax derivation (midaxillary sixth left rib – distal end of sternum) using a 0.5–200 Hz bandpass filter and a 2000 Hz sampling rate. R-R intervals were detected by a custom script (Spike 6.01, Cambridge Electronic Design. UK). After visual inspection of the signal and removal of artefacts, R-R intervalograms were further analysed in the time and frequency domains to provide measures of heart rate variability according to recommendations by the Task force of the European Society of Cardiology [49]; including inter-beat intervals (IBI), standard deviation of normal-to-normal inter-beat intervals (SDNN), the number of successive differences >50 ms between normal-to-normal intervals (NN50), spectral power in low frequency (LF 0,04–0,15 Hz) and high frequency (HF 0,15–0,4 Hz) bands, and the low-to-high frequency power ratio (LF/HF). These variables were retrieved for segments of 136 s during the physical work bouts (three segments in each bout) and while performing the mental task (one segment in each break). After inspection of data, spectral power variables (but not their ratio) were log-transformed to ensure normal distribution.

#### Arterial Blood Pressure

Systolic and diastolic arterial blood pressure (BP) was measured using a non-invasive pressure sensor (NIBP100B-R; Biopac, CA, USA). The sensor was applied just above the radial artery on the left arm, and BP-values based on averaging approximately 15 pulse pressure waveforms were registered. BP was monitored during the entire experiment, and segments of 2 minutes were used for additional analyses; three segments for each physical work bout and one segment for each break.

### Test battery

A test battery (TB, cf. [50]) was performed before, immediately after, and 1 hour after the work period, with 5 minutes of rest between different TB parts (Figure 1b). During the TB, subjects were instructed to sit quietly in their chair to avoid motion artefacts. Instructions for each TB part were orally presented by the examiner and also shown on a computer screen in front of the subject. ECG was monitored throughout the TB (cf. Figure 1b), which otherwise comprised the measurements described below.

#### Maximal voluntary contraction (MVC)

MVC was obtained in bilateral isometric shoulder elevation, encouraging the subject to exert a maximal vertical shoulder “lift” while being fixed to straps connecting the shoulders and the floor. The developed force was read from a dynamometer placed in the right side strap (Somedic Production AB, Sollentuna, Sweden). The best of three attempts interspersed by one minute of rest was saved.

#### Pressure pain threshold (PPT)

PPT was determined over both trapezius muscles using a pressure algometer (Somedic Production AB, Sollentuna, Sweden) applied halfway between C7 and acromion. Pressure was increased at a rate of approximately 50 kPa·s^−1^, and subjects pressed a button as soon as the sensation of pressure turned into pain. The corresponding pressure value was then noted by the examiner. Three measurements were collected from each of the left and right shoulders and averaged to give a PPT for each shoulder.

#### Isometric test contraction

The subject was instructed to raise his straight arms to 90° abduction in the frontal plane and keep them there for one minute. The EMG amplitude of the middle 10 s of this test contraction was retrieved for further analysis. The test contraction requires about 15 percent of the maximal voluntary capacity for an average male subject [50].

### Further data analyses

Descriptive statistics for all variables were expressed as group means and SD between individuals, unless otherwise stated. For data averaged across the three experimental protocols, a pooled SD was calculated as the root of the average variance in the protocols.

In order to investigate the development of fatigue across repeated bouts of physical work, repeated-measure ANOVAs were constructed with work bout (WB, bout 1–6) and MT (MT1, MT2, MT3) as within-subject factors and the order of MT as a between-subjects factor. The effect of WB was modelled as a linear trend using polynomial contrasts. The ANOVA model included interactions between WB and MT. This ANOVA model was resolved for heart rate variability, arterial blood pressure, and amplitude and frequency of EMG (in all cases on results averaged across each WB), and perceived fatigue (using the ratings directly after each WB). If the main effect of MT and/or the interaction MT × WB was found to be significant, a series of post hoc tests were performed, in which the ANOVA model was run on data excluding either MT1, MT2 or MT3.

In order to analyse the immediate recovery effect of the break after each physical work bout, changes in EMG amplitude between the last part of one physical work bout and the initial part of the following bout were noted; i.e. five values in total for each work period. Recovery of cardiovascular variables was assessed by subtracting the value during each work bout from the value during the subsequent break; i.e. six change scores in total for each work period, including values immediately after the last bout. For perceived fatigue, recovery was measured by the difference between ratings early and late in each break; in total five change scores. Each set of change scores was analysed for effects of WB and MT using the ANOVA model described above, including the described post-hoc test procedure.

Recovery during prolonged rest after the entire work period was investigated by comparing the measurements of PPT, MVC, reference contraction EMG amplitude, perceived fatigue, and resting BP and heart rate variability obtained during the test battery before (pre-work), immediately after (post-work0h) and one hour after work (post-work1h). Measurement results were tested using ANOVAs with TB (three levels) and MT (three levels) as within-subject factors, followed by post hoc analyses of pre-work vs. post-work1h values (showing whether responses returned to baseline), post-work0h vs. post-work1h values (showing the extent of recovery during the first hour after work), and pre-work vs. post-work0h (giving a complementary measure of the overall development of fatigue during the work period).

All statistical analyses were performed using SPSS edition 18.0 or later (IBM Inc., Chicago, IL, USA).

## Results

Complete data at the individual level are available on-line, as Dataset S1.

### Exposure and response before and during the initial work bout

Before work, fatigue was, on average, rated 1.3 (SD 0.9) on the CR10 scale, and SOFI ratings were, “*spent”* 0.3, (SD 0.5), “*breathing heavily*” 0.1 (SD 0.3), “*aching*” 0.2 (SD 0.4), “*uninterested*” 1.2 (SD 1.2), and “*sleepy*” 1.7 (SD 1.3). None of these baseline ratings were influenced by the mental task condition in the following work period (all p-values ≥0.3, besides for “sleepy”, p = 0.06). As expected, physiologic variables collected during the test battery preceding work (cf. Table 1) did not differ either between the three mental task conditions (MVC: F = 0.20, p = 0.82; ipsilateral PPT: F = 0.81, p = 0.45; contralateral PPT: F = 0.56, p = 0.58).

**Table 1:** Pre-work baseline values of maximal strength, pressure pain threshold and reference contraction EMG amplitude, and their recovery after work.

Measure	Mental task	Pre-work	Post-work0h	Post-work1h	Main effect TB, p-value	Interaction TB × MT, p-value
**MVC (N)**	MT1	662 (225)	596 (182)	625 (153)	**0.02**	**0.04**
	MT2	642 (132)	608 (153)	622 (127)		
	MT3	645 (170)	634 (208)	582 (161)		
**PPT (kPa)**						
Ipsilateral	MT1	448 (187)	427 (140)	434 (170)	**0.04**	>0.30
	MT2	443 (148)	425 (144)	415 (132)		
	MT3	467 (174)	444 (136)	432 (144)		
Contralateral	MT1	433 (174)	385 (106)	395 (123)	0.21	**0.03**
	MT2	408 (132)	382 (123)	385 (115)		
	MT3	416 (119)	428 (136)	422 (123)		
						
**EMG amplitude, ipsilateral (%RVE** a **)**	MT1	100	113 (13)	106 (8)	**<0.003** b	>0.30^b^
	MT2	100	121 (34)	116 (30)		
	MT3	100	112 (17)	110 (21)		

a : EMG amplitude expressed in percent of the pre-work reference contraction value.

b : Main effect of TB tested using one sample t-tests for post-work0h and post-work1h values pooled across MT against the reference pre-work value (100); interaction effect (TB × MT) investigated by testing for the effect of MT in three separate ANOVAs on results from post-work0h, post-work1h, and the difference between post-work0h and post-work1h.

MVC, Maximum voluntary contraction; PPT, Pressure pain threshold; MT1, MT2, MT3, easy, medium and difficult mental task; TB, Test battery.

Table shows mean (SD between subjects) values of MVC, PPT and reference contraction EMG amplitude in the three test batteries (TB) preceding work, immediately after work and one hour into recovery after work including the three mental tasks (MT1, MT2, MT3). Right-most columns show results of the ANOVA tests (p-values less than 0.05 in bold) for a main effect of TB and an interaction TB × MT.

In the non-fatigued state, i.e. during the first minute of the initial work bout (WB1), the physical work task was performed with a mean ipsilateral trapezius EMG amplitude of 48 (SD 14) %RVE (mean across all subjects and conditions). The mean R-R interval was 794 (SD 92) ms during the first part of WB1 (rest: 856 (SD 93) ms), corresponding to a heart rate of 75.6 beats·min^−1^. Time domain HRV analyses showed a mean SDNN of 58 (SD 20) ms during WB1 (rest: 65 (SD 27) ms), and a NN50 of 17 (SD 14) counts/136s (rest: 28 (SD 20) counts/136s); both confirming the expected decrease in parasympathetic modulation of the heart during work compared to rest [51]. Arterial blood pressure increased as expected during WB1 (Systolic BP 150 (SD 16) mmHg; diastolic BP 85 (SD 10) mmHg) compared to resting values (Systolic BP 134 (SD 14) mmHg; diastolic BP 74 (SD 9) mmHg).

### Fatigue during work bouts

Physical work led to a moderate perceived neck-shoulder fatigue already during the first WB according to ratings on the Borg CR10 scale (mean increase 3.7 (SD 1.3)), and perceived fatigue increased linearly across the whole work period as indicated by a significant WB effect (Table 2; F = 38.4, p<0.001). At the end of the experiment, Borg CR10 ratings had increased to, on the average, 5.8 (SD 1.9). For the SOFI ratings, significant increases across WB was found for *aching* (F = 5.4, p = 0.04), *spent* (F = 44.3, p<0.001), *heavy breathing* (F = 9.1, p = 0.01), and *uninterested* (F = 5.8, p = 0.03).

**Table 2:** Development of perceived fatigue and muscle activity during work.

	MT1	MT2	MT3	WB Main effect p-value	MT Main effect p-value	Interaction WB × MT p-value
**Borg CR10 (0–10**)	2.6 (1.7)	2.2 (1.7)	1.6 (2.1)	**<0.001**	0.29	**0.04**
**SOFI (0–6)**						
*Aching*	0.8 (1.3)	0.8 (1.3)	0.4 (1.7)	**0.04**	0.14	>0.30
*Spent*	1.2 (0.8)	1.2 (0.8)	0.9 (1.3)	**<0.001**	>0.30	0.18
*Sleepy*	0.0 (1.3)	0.4 (1.3)	0.4 (1.3)	0.09	>0.30	>0.30
*Heavy breathing*	0.3 (0.4)	0.4 (0.4)	0.3 (0.8)	**0.01**	>0.30	>0.30
*Uninterested*	0.4 (0.8)	0.3 (1.3)	0.1 (0.8)	**0.03**	>0.30	>0.30
**EMG amplitude (%RVE)**						
Ipsilateral	7.6 (8.2)	5.7 (8.1)	9.5 (8.1)	**<0.001**	>0.30	>0.30
Contralateral	3.0 (8.5)	3.4 (7.2)	4.4 (5.1)	**<0.001**	>0.30	>0.30
**EMG MPF (Hz)**						
Ipsilateral	0.3 (3.0)	−1.2 (2.5)	−1.5 (3.0)	0.08	>0.30	>0.30
Contralateral	3.2 (8.9)	4.8 (8.9)	6.3 (8.1)	**0.003**	>0.30	0.26

MT1, MT2, MT3; easy, medium and difficult mental task.

The difference between work blocks 1 and 6 of perceived fatigue, and of ipsi- and contralateral trapezius EMG variables (means (SD between subjects)) is shown for each of the mental tasks (MT1, MT2, MT3); together with the corresponding results of the ANOVA tests, based on all six work blocks, for main effects and interaction of work bout (WB) and mental task (MT) condition (p-values less than 0.05 in bold).

**Figure 2:**
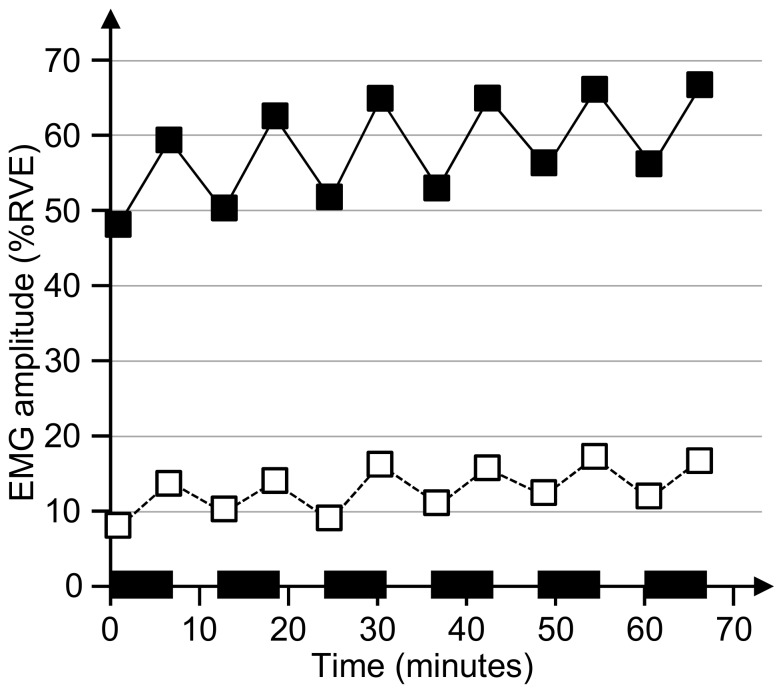
Trapezius EMG amplitude during the work period. EMG amplitude during the first and last minute of each of the six work bouts; mean across subjects and mental task conditions. X-axis shows time after commencing work; black zones mark physical work bouts. Filled and empty symbols with full-drawn and dashed line: ipsi- and contralateral trapezius EMG, respectively.

A significant linear increase across work bouts was found for the average ipsilateral trapezius EMG amplitude during bouts (F = 32.1, p<0.001), while MPF decreased (F = 3.6, p = 0.08). Trapezius EMG amplitude also increased at the contralateral side, but remained at considerably lower levels (Table 2, Figure 2).

Heart rate, calculated from IBI, decreased significantly with WB (F = 7.7, p = 0.014), while heart rate variability increased, as measured both by SDNN (F = 4.4, p = 0.05) and NN50 (F = 8.2, p = 0.01). Neither frequency-related variables of heart rate variability nor BP were significantly affected by WB.

Pre-post differences of test battery measurements confirmed that the work period led to fatigue (Table 1); the reference contraction EMG amplitude was, on average, 15 (SD 22) %RVE higher after work than before (F = 11.2, p = 0.004), and MVC had declined from 649 (SD 176) N to 610 (SD 185) N (F = 6.0, p = 0.03). The difficulty of the mental task did not have any significant main effect on perceived fatigue (Table 2). An interaction effect (WB × MT) was, however, found for the Borg CR10 ratings (F = 4.4, p = 0.04). Post-hoc tests showed that perceived muscle fatigue developed at a lower rate for the most difficult memory tasks than for the easy task (MT3 vs MT1: F = 4.8, p = 0.04.

The amplitude and frequency of EMG was not significantly influenced by MT (Table 2), and interactions between WB and MT were not found either. For heart rate variability and blood pressure during work, main effects of MT were observed in SDNN (F = 8.6, p = 0.01), NN50 (F = 5.8, p = 0.03), systolic BP (F = 4.9 p = 0.05) and diastolic BP (F = 7.6, p = 0.02). Post-hoc tests showed that MT1 was associated with lower heart rate variability than MT3, while blood pressure was elevated in the experiment with MT2 compared to MT3. No significant WB × MT interaction effects were found for these variables.

### Recovery during breaks

Perceived neck-shoulder fatigue was significantly reduced during the breaks, indicating a rapid recovery after each WB, typically from “high” fatigue to “weak” fatigue at the Borg CR10 scale (F = 69.8, p<0.001). Recovery in CR10 ratings did not differ between the mental tasks (F = 1.0, p = 0.40). A tendency to higher alertness was, however, seen in breaks during the difficult mental task (MT3) condition, since the increase in SOFI rating of *sleepy* during those breaks was smaller than during breaks in the other two mental task conditions (F = 7.9, p = 0.01).

Trapezius EMG amplitude recovered between the last part of one WB and the first part of the next one (Figure 2). The extent of recovery in the ipsilateral EMG amplitude depended on WB (F = 2.9, p = 0.04); recovery diminishing slightly with time. The difficulty of the mental task had no significant effect on recovery of neither ipsilateral nor contralateral EMG (MT main effects: F = 0.97, p = 0.39 (ipsi) and F = 2.34, p = 0.11 (contra); interactions WB × MT: F = 0.39, p = 0.93 (ipsi) and F = 1.0, p = 0.44 (contra)).

**Figure 3:**
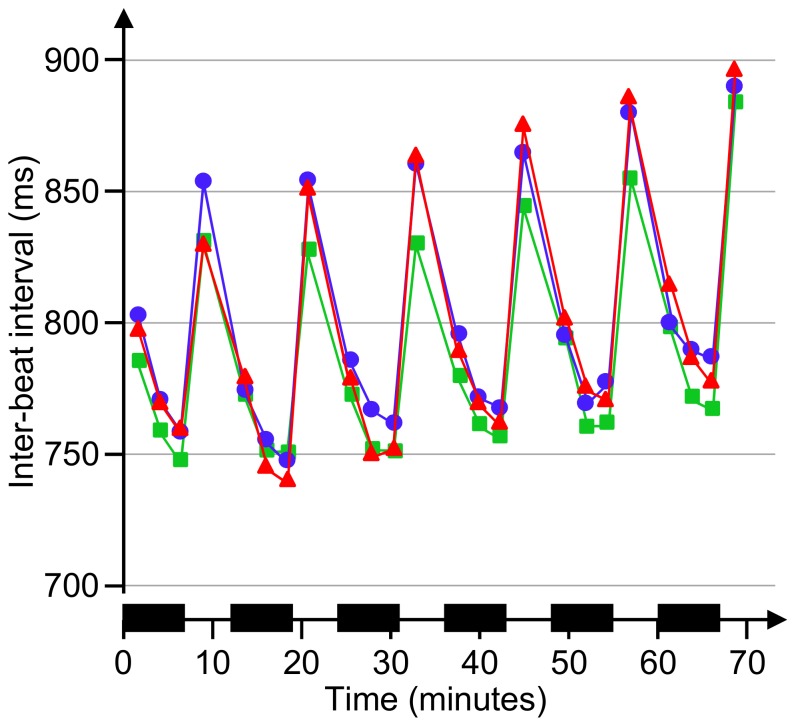
Heart rate during the work period. Heart rate (measured by inter-beat ECG intervals) during work and breaks in the easy (MT1, green squares), medium (MT2, blue circles), and difficult (MT3, red triangles) mental task protocols. X-axis shows time after commencing work; black zones mark physical work bouts.

Heart rate variability increased during breaks (Table 3). Enhanced recovery of heart rate and NN50 was found for the MT3 condition (Figure 3), as indicated by significant interactions between WB and MT (F = 8.6, p = 0.009; and F = 6.8, p = 0.02). Similarly, the LF/HF ratio during breaks was decreased for MT3 compared to MT1 (main effect of MT; F = 6.7, p = 0.02), mainly due to a larger HF content (F = 10.3; p = 0.006). Recovery of arterial blood pressure during the breaks did not show any dependence on MT.

**Table 3:** Recovery of heart rate, heart rate variability, and blood pressure during the break after each bout of physical work (WB1-WB6), stratified by mental task (MT1, MT2, MT3); mean (SD between subjects)^a^.

	Mental task	WB 1	WB 2	WB 3	WB 4	WB 5	WB 6
**Heart Rate**							
IBI (ms)	MT1	84 (59)	79 (42)	79 (47)	88 (49)	93 (57)	117 (58)
	MT2	96 (52)	107 (66)	99 (60)	98 (49)	103 (56)	103 (59)
	MT3	70 (58)	111 (65)	110 (63)	112 (74)	116 (60)	119 (58)
**Heart Rate Variability**							
NN50 (counts)	MT1	8 (18)	8 (14)	6 (11)	5 (15)	11 (13)	16 (15)
	MT2	12 (22)	11 (21)	12 (18)	8 (13)	15 (16)	15 (21)
	MT3	8 (17)	13 (19)	10 (18)	14 (18)	15 (12)	16 (15)
SDNN (ms)	MT1	11 (32)	16 (24)	10 (20)	13 (20)	21 (19)	28 (32)
	MT2	16 (25)	18 (21)	27 (34)	20 (22)	31 (40)	26 (33)
	MT3	7 (26)	19 (18)	14 (26)	27 (37)	21 (24)	26 (27)
ln LF (ms^−2^)	MT1	−0.4 (1.0)	0.5 (0.9)	0.3 (1.0)	0.3 (0.9)	0.5 (0.7)	0.6 (0.8)
	MT2	−0.3 (1.0)	0.2 (0.9)	0.4 (0.9)	0.5 (0.9)	0.8 (1.1)	0.6 (0.8)
	MT3	−0.3 (1.2)	0.6 (1.0)	0.1 (0.8)	0.4 (1.0)	0.4 (0.9)	0.4 (0.9)
ln HF (ms^−2^)	MT1	0.5 (1.1)	0.6 (0.9)	0.4 (1.0)	0.5 (1.0)	0.8 (0.6)	0.9 (1.0)
	MT2	0.6 (1.3)	0.7 (1.0)	0.8 (1.2)	0.8 (1.2)	0.9 (1.0)	0.8 (1.0)
	MT3	0.6 (1.0)	1.0 (0.9)	0.6 (0.8)	0.8 (1.0)	0.8 (0.6)	0.9 (1.1)
LF/HF ratio	MT1	−4.9 (6.6)	−1.9 (6.8)	−1.0 (5.1)	−2.7 (6.8)	−2.9 (8.4)	−2.6 (5.0)
	MT2	−5.0 (4.9)	−2.4 (4.5)	−2.0 (5.0)	−3.9 (9.9)	−2.2 (5.2)	−1.7 (4.8)
	MT3	−4.2 (4.1)	−2.1 (3.2)	−3.3 (5.2)	−3.0 (4.6)	−3.0 (6.6)	−2.5 (4.5)
**Blood Pressure**							
Systolic (mmHg)	MT1	−12.8 (9.6)	−15.2 (9.8)	−9.7 (9.1)	−13.4 (9.1)	−13.2 (10.0)	−13.3 (10.1)
	MT2	−14.3 (9.0)	−12.1 (14.2)	−11.8 (12.4)	−16.7 (9.0)	−14.8 (5.8)	−14.0 (7.1)
	MT3	−10.4 (7.9)	−11.1 (9.9)	−11.7 (12.3)	−12.9 (10.9)	−13.6 (9.5)	−9.8 (15.4)
Diastolic (mmHg)	MT1	−7.1 (5.8)	−11.0 (8.6)	−6.7 (8.1)	−7.8 (6.3)	−9.9 (7.4)	−9.7 (6.0)
	MT2	−10.5 (6.5)	−9.0 (7.0)	−6.5 (8.3)	−10.3 (6.8)	−9.4 (4.6)	−9.2 (5.4)
	MT3	−7.5 (6.2)	−8.2 (6.7)	−7.5 (7.4)	−7.3 (6.7)	−10.0 (7.5)	−8.1 (8.8)

a : scores were calculated by subtracting the value during work from the value during the subsequent break.

IBI, inter-beat interval. For Heart rate variability metrics, see methods section. MT1, MT2, MT3; easy, medium and difficult mental task.

### Recovery after work

**Figure 4:**
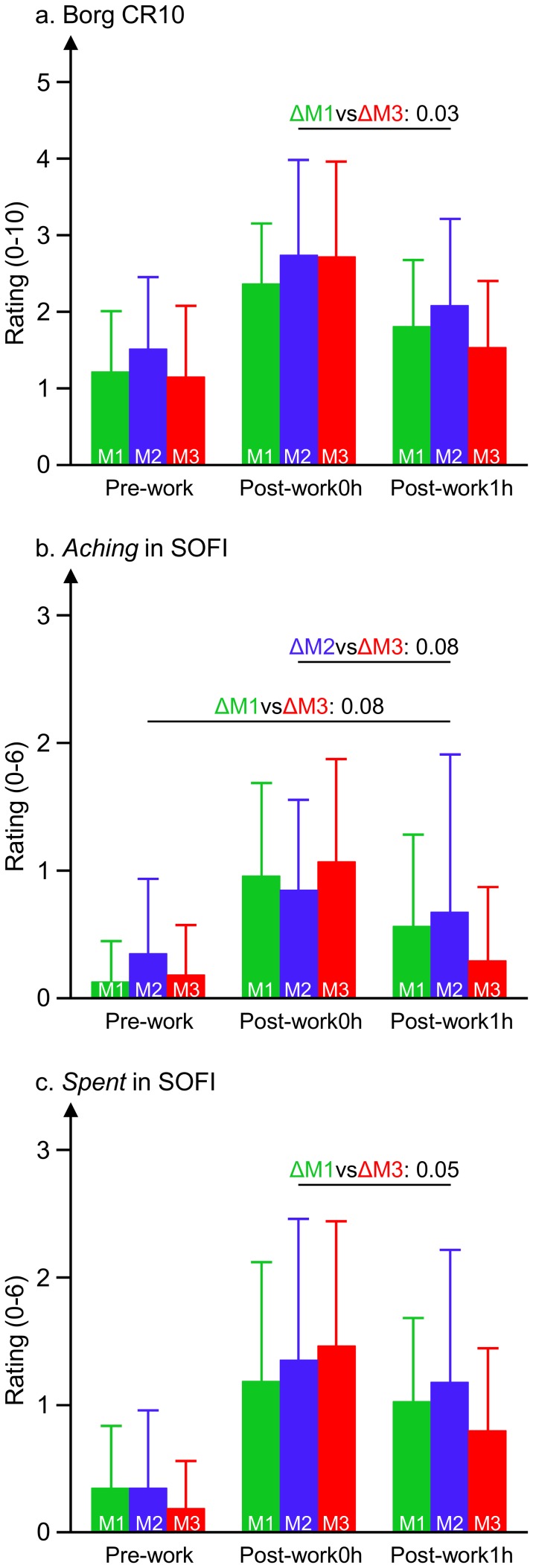
Ratings of perceived fatigue before, immediately after, and one hour after the work period. Perceived fatigue rated on, (a) the Borg CR10 scale, (b) *aching* in SOFI, and (c) *spent* in SOFI; before and after work in the easy (MT1, green), medium (MT2, blue) and difficult (MT3, red) mental task protocols. Mean values; bars illustrating SD between subjects. Text and numbers above columns mark differences in recovery between mental task conditions reaching a p-value less than 0.10. As an example, for *spent* the change from post-work0h to post-work1h differed between M1 and M3 at a p = 0.05 level of significance.

As expected, perceived fatigue diminished considerably during the first hour after the work period (Figure 4). Recovery of Borg CR10 ratings differed depending on the preceding mental task, and was most pronounced after the most difficult cognitive task, MT3 (F = 7.8, p = 0.01). SOFI ratings also recovered after work (p<0.05), and trends suggesting an MT-by-time interaction were seen for *aching* (F = 3.9, p = 0.07) and *spent* (F = 3.35, p = 0.05), with recovery one hour after the work bout being most pronounced for the difficult mental condition MT3.

MVC was significantly reduced immediately after the experiments (Table 1; F = 5.9, p = 0.03) and did not recover fully during the hour after work (F = 4.8, p = 0.04). PPT on the ipsilateral side also decreased after the experiment (F = 4.8, p = 0.04). The extent of recovery after the experiment differed depending on the preceding mental task (MVC (F = 5.3, p = 0.04); contralateral PPT (F = 5.4, p = 0.03)). For PPT, recovery was slower (or even indiscernible) after MT3, while MVC was higher immediately after MT3 than after MT1 and MT2, but did then not recover during the following hour (Table 1). The reference contraction EMG amplitude had recovered significantly after one hour towards the value preceding work (F = 5.1, p = 0.04) (Table 1), and recovery did not differ depending on the mental condition. All cardiovascular indices were increased above baseline values immediately after work, and remained increased during the first hour of rest, yet to a similar extent irrespective of the preceding MT (values not shown).

## Discussion

Motivated by our interest in fatigue and recovery in an occupational context, we selected a physical task for this experiment, which simulated repetitive manual handling of weights consistent with those occurring in many industrial settings, for instance in manufacturing [52]–[54] or slaughterhouses [55], [56]. Fatigue and disorders in repetitive work is a major concern in occupational epidemiology, and the need to intervene has been emphasized in several reviews [57]–[60]. Our repetitive task did lead to pronounced, if not extreme, fatigue, as confirmed by subjective ratings (Table 2) and changes in the electromyographic (EMG) signal from the upper trapezius (Table 2) conventionally used as a sign of decreased force-generating capacity of the exercising muscles [47]. Also, subjects changed their motor strategy in the course of the work period, as shown in a previous publication of motor control in the physical task [41]. As expected from previous studies of intermittent work involving the arms [50], [61], fatigue increased during each of the physical work bouts, and recovered partly, though not fully, in the subsequent break, such as illustrated by the EMG pattern in Figure 2. Also, signs of fatigue persisted one hour after work, as reflected in decreased maximal shoulder elevation strength (Table 1). This general pattern applied irrespective of the cognitive task performed during the breaks, even though maximal strength was reduced more one hour after the most difficult task, MT3, than after the two other conditions, MT1 and MT2.

We found indications that autonomic activity differed depending on the difficulty of the mental task; the most demanding task was associated with better recovery of heart rate and heart rate variability than the easy task, reflecting an accelerated return towards more parasympathetic cardiac activity (Table 3). Also, perceived fatigue developed slower during the protocol with the most demanding task (Table 2), and recovered faster after work (Figure 4), while muscle activity (as reflected by EMG) was not sensitive to the cognitive demands. One explanation that the most difficult mental task condition was associated with the largest parasympathetic involvement could be that the task was experienced to be so difficult that subjects gave in and lost motivation. However, this is not a likely explanation, considering that performance was consistently high (on average 83% correct answers) throughout the experimental session.

While these effects of a mental task on recovery after physical work were not very strong, and to some extent ambiguous, they do support the reports by Asmussen & Mazin [36] and Stock et al. [38] that a cognitive effort may enhance recovery compared to a passive break. This consistency is a remarkable result, considering that these two studies used physical task paradigms differing substantially from ours. In the Asmussen & Mazin study, subjects performed repeated submaximal force exertions using the flexors of the elbow or the middle finger till exhaustion, while Stock et al. had their subjects perform 50 fast and consecutive maximal isokinetic knee extensions. Thus, both studies used experimental tasks requiring a maximal effort, in terms of endurance and force, respectively, while our physical task required 5–10% of the maximal force-generating capacity, as judged from the average trapezius EMG amplitude in the first work bout, and was far from exhaustive. In keeping with their experimental tasks, both Asmussen & Mazin and Stock et al. measured fatigue using maximal performance metrics, while we applied a much wider selection of physiologic and psychophysical variables of relevance to central and peripheral fatigue.

Both Asmussen & Mazin and Stock et al. used a challenging, yet manageable mental arithmetic task during their “active” breaks, but did not explicitly assess neither intensity nor performance, beyond the instruction used by Stock et al. to “correctly answer as many problems as possible” in a 3-minute period. In our study, the mental task was strictly controlled in terms of speed and difficulty, and we kept track of the subjects' performance, i.e. the proportion of correct answers. As a “passive” reference condition, both Asmussen & Mazin and Stock et al. used quiet rest, while we used a standardized and very easy mental task, requiring subjects to devote their attention to the same cognitive activity which was then modified with respect to difficulty in the other experimental conditions. Any difference in physiologic response between the three mental conditions will therefore reflect a specific effect of changes in demands on the working memory, rather than an unspecific effect of mental attention, had the subjects been left in uncontrolled passive rest as the reference condition.

The studies by Asmussen & Mazin and Stock et al. presented evidence that a likely explanation why a mental task could influence recovery is that it changes the balance between inhibition and activation of brain centres involved in central fatigue [36], [39]. The latter explanation is an extension of theories presented about a century ago by Sechenov (cited in [18]), who noted that both physical and mental “diverting activities” after a period of physical work seemed to improve performance in a subsequent work bout. Substantial research has confirmed that fatigue caused by muscle activity is, indeed, a complex result of both peripheral and central components [22], [23], and that cognitive activity does change the activity in brain regions known to also be activated during a fatiguing physical task [62]–[64]. Our results corroborated that a possible recuperating effect of a demanding cognitive activity in short breaks between bouts of physical work, as well as during a longer rest period after work may, indeed, reside in the central nervous system, even though we did not explicitly measure brain activity using, e.g. electroencephalography or functional resonance imaging. The parasympathetic branch of the autonomic nervous system seemed to be involved in this modulation, as suggested even by studies showing that brain regions associated with parasympathetic activation are engaged during both mental and physical tasks [65], [66]. The effect of a cognitive activity may thus be to change autonomic balance towards a larger parasympathetic involvement during recovery, rather than to promote an increased arousal as implied by Sechenov's theories. However, the modulating pathways remain to be verified by direct measurements of central nervous system activity.

While the available literature, including our study, suggests that the positive effect of a mental task on recovery may be found across a wide range of physical tasks, it appears reasonable to assume that the effect will be particularly pronounced in situations where, 1) fatigue caused by the physical task has a strong central component, 2) the physical work per se does not impose any strong cognitive demands, 3) the cognitive task is sufficiently demanding to activate the brain, but not so demanding that it leads to additional central fatigue or stress. Also, the specific effect of the mental task can be expected to differ between individuals according to their working memory and capacity for physical work. If this hypothesis is valid, the effect of a mental break should, for a particular individual, be most pronounced when performing a stereotyped physical task (i.e. a task with an automated motor control, [67] chapter 4) at a moderate intensity, and combining it with a mental task which is challenging but manageable, in a rotation scheme with reasonably frequent shifts (since frequent breaks lead to less fatigue than “rare” breaks [50]). Further research may examine this expectation, and the proposed occurrence of individual differences related to physical and mental capacity.

In an occupational interpretation, the idea that mental tasks in-between physical work bouts may be more beneficial to performance than passive breaks adds to a vivid discussion of the possible benefits of initiatives promoting variation, in particular in repetitive or constrained physical work. While passive rest breaks do, indeed, lead to a remarkable recovery of fatigue following from strictly controlled isometric and isotonic contractions [1]–[5], they show less encouraging effects when introduced in real occupational settings [9]. One reason may be that the proportion of a work day that can realistically be devoted to (non-productive) rest breaks is too small for the breaks to have any notable physiologic effect; intervention studies have, in general, implemented breaks corresponding to 5-10% of the total time at work [68], [69]. This prompts the idea of identifying productive tasks that allow recovery from physical work, and that can feasibly be practiced for sufficient proportion of the work time to have a clear effect. In our study, about 30 percent of the “work cycle” was devoted to the mental task, and in many settings, even in industrial assembly, it may be feasible to introduce productive cognitive activities, such as administrative work [70]. Also, the suggested beneficial effect of mental activities may be realized by offering workers opportunities for cognitive stimulation during scheduled breaks off work.

In an even wider interpretation, alternating physical and mental tasks may be viewed as an idea for job rotation. The general aspiration in job rotation is to identify a combination of tasks that can be performed without loss in productivity or substantial fatigue (and, possibly, less risk of developing musculoskeletal disorders), compared to performing one of the component tasks alone [9], [71], [72]. While job rotation has generally been discussed in the context of mixing physical work tasks [17], [73]–[78], and while effects of physical variation on fatigue and motor control are, indeed, promising [9], [79], [80], the present concept of alternating between physical and mental tasks widens job rotation to even include productive tasks that require cognitive efforts while posing very small biomechanical demands.

In conclusion, the results of the present study support a few previous investigations suggesting that mental tasks interspersed between bouts of fatiguing physical work may provide recovery more effectively than passive breaks, even if, in the present study, the benefits were moderate, mainly reflected in different cardiovascular responses and perceived fatigue, and to some extent contested by the maximal strength response. Thus, our results encourage further research, both in a basic physiological context and motivated by questions of occupational relevance, such as which combinations of physical and mental tasks that may represent particularly attractive mutual effects; which time patterns of alternation that lead to the better results in terms of physiologic responses and acceptability; and which personal traits that may influence the outcome of alternating between physical and mental tasks.

## Supporting Information

Dataset S1 **Complete data at the individual level.**
(XLSX)
